# Conjugated Aggregation-Induced Fluorescent Materials for Biofluorescent Probes: A Review

**DOI:** 10.3390/bios13020159

**Published:** 2023-01-19

**Authors:** Zheng Wang, Ji Ma, Changlin Li, Haichang Zhang

**Affiliations:** Key Laboratory of Rubber-Plastics of Ministry of Education/Shandong Province (QUST), School of Polymer Science and Engineering, Qingdao University of Science and Technology, 53-Zhengzhou Road, Qingdao 266042, China

**Keywords:** aggregation-induced emission (AIE), fluorescent probes, labeling, cells, fluorescent

## Abstract

The common fluorescent conjugated materials present weak or quenching luminescent phenomena in the solid or aggregate state (ACQ), which limits their applications in medicine and biology. In the last two decades, certain materials, named aggregation-induced emission (AIE) fluorescent materials, have exhibited strong luminescent properties in the aggregate state, which can overcome the ACQ phenomenon. Due to their intrinsic properties, the AIE materials have been successfully used in biolabeling, where they can not only detect the species of ions and their concentrations in organisms, but can also monitor the organisms’ physiological activity. In addition, these kinds of materials often present non-biological toxicity. Thus, AIE materials have become some of the most popular biofluorescent probe materials and are attracting more and more attention. This field is still in its early infancy, and several open challenges urgently need to be addressed, such as the materials’ biocompatibility, metabolism, and so on. Designing a high-performance AIE material for biofluorescent probes is still challenging. In this review, based on the molecular design concept, various AIE materials with functional groups in the biofluorescent probes are introduced, including tetrastyrene materials, distilbene anthracene materials, triphenylamine materials, and hexaphenylsilole materials. In addition, according to the molecular system design strategy, the donor–acceptor (D-A) system and hydrogen-bonding AIE materials used as biofluorescent probes are reviewed. Finally, the biofluorescent probe design concept and potential evolution trends are discussed. The final goal is to outline a theoretical scaffold for the design of high-performance AIE biofluorescent probes that can at the same time further the development of the applications of AIE-based biofluorescent probes.

## 1. Introduction

There are various essential anions and metal cations in the human body, such as F^−^, Ca^2+^, Zn^2+^, and so on. An imbalance of ions in cells can lead to diseases. The detection of these ions in the living cells might indicate whether there has been a pathological change in cells, which can play a crucial role in monitoring people’s physical condition. Recently, more and more studies have reported on biosensor materials. However, traditional organic fluorescent materials have bright fluorescence levels when they are in a high dispersion state in a good solvent. While in the aggregated state, the fluorescence becomes weak or even quenching, which is called “aggregation-induced quenching” (ACQ). Scholars have adopted many methods to solve the ACQ phenomenon, such as reducing the doping concentration of the fluorescent material, and in turn reducing the aggregation degree. However, the aggregation of molecules is a spontaneous process in thermodynamics, which is difficult to inhibit using various physical methods.

The organic materials with strong luminescence in the solid or aggregated state are more favorable, since the fluorescence can be easily detected using equipment. In 2001, Tang’s group [1] found that hexaphenylsilole (HPS) does not emit light in the highly dispersed state, but exhibits extremely shining fluorescence under the aggregated state, which presents totally unusual optical phenomena. The authors found that the six benzene rings in the molecule can rotate or vibrate very freely, so that the energy transferred to them by ultraviolet light can be consumed via rotation or vibration without fluorescent radiation in the dispersed state. 

**Figure 1:**
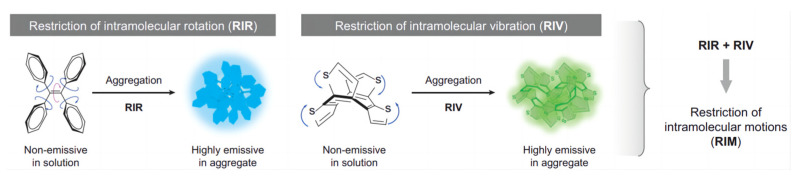
Tetraphenylethene (TPE) is non-emissive when dissolved but becomes emissive when aggregated due to the restriction of intramolecular rotation (RIR), restriction of intramolecular vibration (RIV), and restriction of intramolecular motion (RIM). Adapted with permission from [2].

However, under the aggregate state, through the restriction of intramolecular rotation or vibration [2] (RIR, RIV, and RIM, Figure 1), the system cannot undergo non-radiative decay. Thus, the energy needs to find another way to dissipate in terms of producing bright fluorescence. The author named such materials aggregation-induced emission (AIE) materials. It seems that the restriction of the intra-molecular motion is a simple and useful method for developing AIE materials. Later, Yang’s group [3] designed a compound using the phenyl groups to substitute the 9,10-position of anthracene, which exhibits a strong blue emission under the aggregate state. Consequently, more and more different AIE materials have been developed by the scientific community, such as tetrastyrene materials [4,5,6,7,8,9], distilbene anthracene materials [10,11,12,13,14], triphenylamine materials [15,16,17,18,19,20], hexaphenylsilole materials [21,22,23,24,25], pyrene materials [26,27,28,29,30], and so on. These are important functional groups, which have recently found wide applications not only for bioimaging but also for detecting other environmental responses. Among these kinds of materials, the most investigated application is in biofluorescent probes for use in organisms. 

AIE materials such as biofluorescent probes are widely used to detect the contents of various ions in organisms, such as Zn^2+^, Hg^+^ Ca^2+^, and so on [31,32,33,34], which can be used to characterize the health of an organism and predict disease. In addition, these kinds of materials are also used in drugs to label the diseased organism for targeted drug release [35,36,37]. Based on the requirements of biology and medicine and the development of computational science [38,39,40,41,42,43,44,45], researchers have developed a number of biocompatible AIE materials for fluorescent probes and monitored various ions in living organisms, which is very meaningful. However, to obtain such high-performance AIE materials as fluorescent probes, the materials should exhibit not only strong fluorescence under the aggregated state but also biocompatibility, non-toxic properties, metabolic effects, and so on. This field is still in its early infancy and several open challenges urgently need to be addressed. 

Many researchers have combined electron-rich and electron-deficient units to form donor–acceptor (D-A) fluorescent probes [46,47,48]. The D-A strategy can adjust the material emission color and wavelength in the optical spectrum, meaning that the materials can probe the cells more easily and clearly. Moreover, introducing a hydrogen-bonding system in the molecules is another useful and simple molecular design strategy for molecular conformation control [49,50,51]. For one thing, hydrogen bonds can reduce the intra-molecular motion in the aggregate state, which can lead to a high emission intensity. Additionally, hydrogen bonds can boost the links or inter-molecular interaction between fluorescent probe molecules. Therefore, the recognition of specific molecules or ions will be strengthened by the restriction of the diffusion phenomenon.

In this review, firstly different kinds of AIE materials with functional groups such as fluorescent probes are reviewed, including tetrastyrene (TPE) materials, distilbene anthracene (DSA) materials, triphenylamine (TPA) materials, and hexaphenylsilole (HPS) materials. Although some articles have reviewed the AIE materials in the biosensors, to the best of our knowledge, only a few articles have discussed the D-A system and hydrogen bonding association in the AIE biosensors. [52,53,54]. Herein, according to the molecular system design strategy, the D-A system and hydrogen-bonding AIE materials used as fluorescent probes are system introduced. In the end, the future outlook is highlighted. The final goal is to outline a theoretical scaffold for the design of high-performance AIE fluorescent probes that can at the same time further the development of the applications of AIE-based fluorescent probes.

## 2. AIE Material Design Strategy Based on the Functional Groups

Material design concepts play a key role in obtaining high-performance AIE materials. So far, for conjugated AIE materials, the most acceptable strategy is a restriction of the intra-molecular motion. To further increase the emission intensity, the most used method is enhancing the overlap of the π-electron while forming a conjugate system of polyatomic orbitals. However, this method is usually a double-edged sword. Because the π-conjugation extension can cause a high fluorescence efficiency, with the increasing conjugation, the π-conjugation system might induce better π–π stacking interactions [55]. Therefore, when designing the AIE molecules in fluorescent probes of the conjugated system, the rationality of the conjugated system should be considered. 

**Figure 2:**
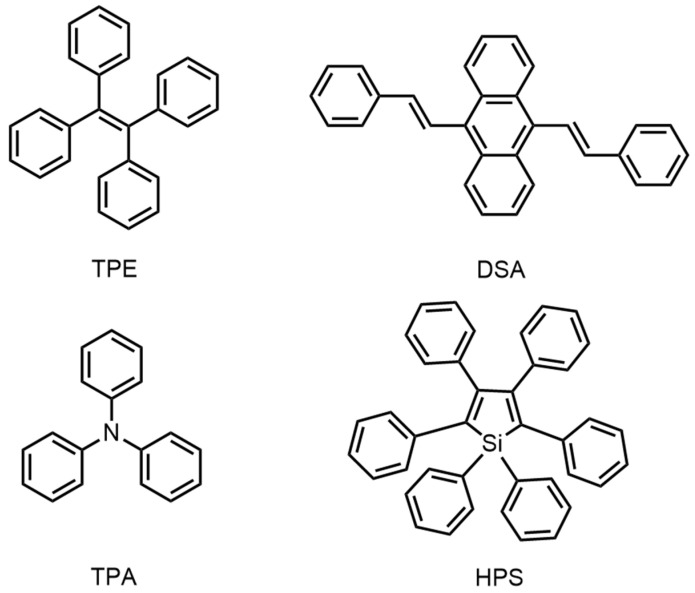
Chemical structures of TPE, DSA, TPA, and HPS.

On this basis, scientific researchers have developed many AIE systems in fluorescent probes with high luminous efficiency in the aggregated state. However, most of these fluorescent probes contain some functional cores, through which other units can be substituted to reduce the intra-molecular motion in the aggregate state. These kinds of cores are usually triphenylamine (TPA) or hexaphenylsilole (HPS). In addition, introducing some functional groups, such as tetraphenylethylene (TPE) and biphenylvinyl anthracene (stilbene anthracene, DSA), could also help in obtaining the AIE materials (Figure 2). 

Tetrastyrene and its derivatives belong to one of the most used and studied AIE materials, which was first found by Tang’s group [56]. In the TPE system, the benzene ring is connected to a single bond of vinyl and the rotational potential resistance in space is small. This means that the benzene ring can rotate or vibrate very freely in a dispersed state, while the rotation or vibration of the benzene ring is restricted in the aggregated state in terms of producing bright fluorescence. Recently, Jianxin Guan et al. [57] systematically investigated the AIE mechanism regarding TPE-based materials. They passed the real-time structural evolution and dynamics of the electronically excited state with frequency and polarization-resolved ultrafast UV/IR spectroscopy as well as theoretical calculations. In addition, the luminescence mechanism of the TPE-based AIE materials was conducted in-depth. The author found that the TPE-based materials pass through the cone intersection in the dispersed state; meanwhile, the electron excitation energy quickly becomes non-radiated via attenuation. However, in the aggregate state, these molecules cannot pass through the cone intersection, and the electron excitation energy can be preserved for a long time. This results in a slow transfer of energy or charge between molecules and avoids the ACQ of the combined results. Whether or not the materials pass through a tapered intersection plays a crucial role in determining the ratio of radiated to non-radiative transitions.

**Figure 3:**
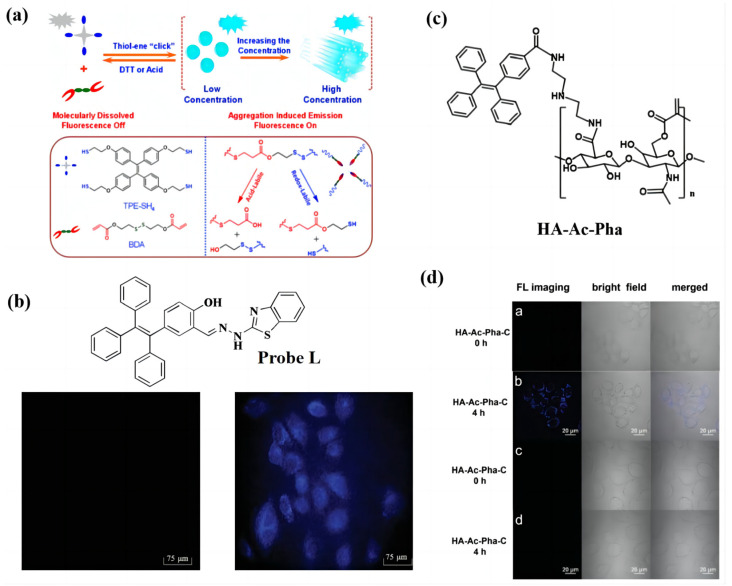
(**a**) Molecular structure of TPE-SH_4._ (**b**) Molecular structure of probe L and Fluorescence image of HeLa cells incubated with pure L (10 μM) and L + Zn^2+^ (100 μM). Adapted with permission from [60]. (**c**) Molecular structure of HA-Ac-Pha. (**d**) LSCM images of cells, which were excited by a 405 nm laser. The HeLa cells were incubated with 40 μg mL^−1^ of HA-Ac-Pha-C for 0 h and 4 h. The L929 cells were incubated with 40 μg mL^−1^ of HA-Ac-Pha-C for 0 h and 4 h. Adapted with permission from [61].

Based on the above AIE mechanism, a series of novel TPE-based materials were designed and used in fluorescent probes with good performance. Sheng-yu Shi et al. [58] used a thiol one-click reaction to synthesize the thiol TPE (TPE-SH_4_), through which a three-dimensional gel network was built (Figure 3a). The three-dimensional gel network had the properties of acid- and redox-switchable aggregation-induced emission characteristics from TPE-SH4 and bis(2-acryloyloxyethyl) disulfide (BDA) via a thiolene click reaction. Thus, the fluorescence emission of the polymer gels can be observed in the absence of dithiothreitol and trifluoroacetic acid, which results in this system’s potential application value in fluorescence sensing. 

Bo Song’s group [59] reported on nanowires contained TPE functional groups with AIE properties. The nanowires were successfully applied in labeling HeLa cells with high-viability and high-contrast fluorescence images. Therefore, the nanowires showed potential in the field of biological imaging. Besides the cells, the TPE-based materials could also detect the ions. Wang et al. [60] synthesized a small molecule, named **L**, containing the TPE group and the functional units, such as hydroxyl and amide, which can react with the Zn^2+^. The small amount of probe **L** exhibited almost no fluorescence in the HeLa cells. However, it exhibited strong blue fluorescence in the RPMI-1640 solution containing 100 μM Zn^2+^ and incubated for 15 min (Figure 3b). The results indicated that probe **L** has good membrane permeability and can be used for the imaging of Zn^2+^ in living cells. The pH is a significant factor in the probe, which should be considered by researchers. Wang et al. discussed the pH effect on probe **L** [60]. Other studies also reported on the use of TPE-based materials for Zn^2+^ sensors in living cells with good performance. For fluorescent materials, they should be stable in living cells. However, this property is often ignored in most reported studies. Li et al. [61] presented a TPE-based AIE nanoparticle as a fluorescence material with good stability, even in living cells after one week (Figure 3c). In addition, when the materials were incubated with Hela cells, the cells were colorless at the beginning, and then they exhibited bring blue fluorescence after 4 h. However, when they were incubated with the L929 cells, it was found that the cells were colorless at all times (Figure 3d). These nanoparticles can be used to image cancer cells, which means the materials have the potential application for in tumor detection. The further development of TPE-based AIE fluorescent materials is important. 

**Figure 4:**
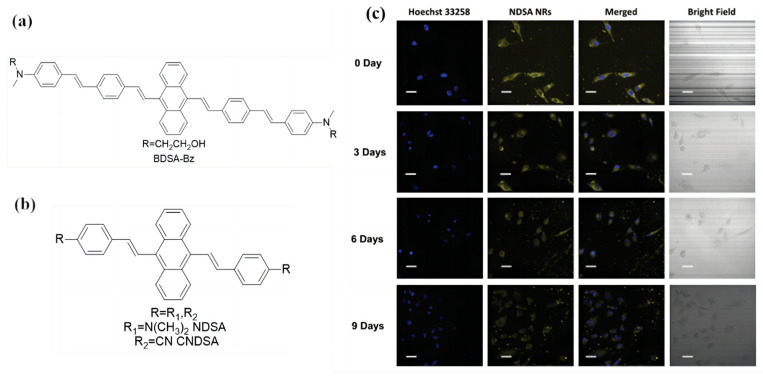
(**a**) Molecular structure of BDSA-Bz. (**b**) Molecular structures of NDSA and CNDSA. (**c**) Long-term cell tracing CLSM images of NDSA NRs incubated at 37 °C for 4 h and then subcultured for fixed time intervals, including day 0, day 3, day 6, and day 9. Scale bar: 20 mm. Adapted with permission from [68].

Recently, besides the functional TPE groups, the stilbene anthracene units have also been popular in the design of AIE materials. In the DSA system, the vinyl benzene ring is connected to a single bond of anthracene. The rotational potential resistance in space between the two groups is small. Thus, the conformation of DSA molecularly can be distorted freely in a dilute solution, which is restricted in the aggregated state. This might result in DSA-based molecules with AIE properties. In 2006, Prasad’s [62] research group first found the phenomenon of the enhanced fluorescence of anthracene compounds in the aggregate state (Figure 4a). The authors analyzed the DSA-based materials’ AIE mechanism. They found that the internal steric hindrance after conjugation can cause a partially distorted structure, which results in the DSA molecules exhibiting a strong AIE phenomenon, similar to the TPE molecules; this was the prelude to DSA-based AIE material research [63,64,65,66,67]. 

In the past few years, numerous novel DSA-based materials have been designed; meanwhile, various applications have also been developed. Among these, the most used application is for fluorescent sensors in living cells. Han et al. [68] prepared two symmetrical DSA-based AIE molecules, namely NDSA and CNDSA fluorescent nanoparticles, as shown in Figure 4b. The authors found great cellular uptake and long-term (even after 9 days) bioimaging results for NDSA and CNDSA in A549 cells with strong blue fluorescence. For the sensors, the materials should exhibit non-toxic properties as well as good biocompatibility (Figure 4c). In this work, the authors found that more than 90% of the A549 cells were alive with concentrations of up to 30 μM of NDSA and CNDSA for 24 h, which means that the cytotoxicity of NDSA and CNDSA toward A549 cells is negligible. Additionally, Han et al. used the ultrasound-aided nanoprecipitation method to enhance the stability of fluorescent sensors. This method has been used for many excellent probes. This research revealed that DSA-based materials have potential applications in sensors. 

The TPE and DSA groups are often the side chain in the design of AIE molecules. However, introducing some functional cores can also help in obtaining good AIE materials, such as triphenylamine and hexaphenylsilole. The two molecules belong to a non-planar helical molecule. The core is connected by a single C-C bond to the multi-side benzene rings with a large torsional angle between these side benzene rings and the central core. As previously described, when the molecules are in a dispersed state, the benzene ring rotates around the core through a single bond in the isolated molecule. The light energy is converted into thermal energy under a disappearing state due to molecular vibration or rotation. However, the movement of the phenyl is limited under the aggregate state, which means that light cannot be converted into thermal energy; meanwhile, the AIE phenomenon occurs. Thus, these two molecules are often designed as the core to obtain high-performance AIE biosensors.

**Figure 5:**
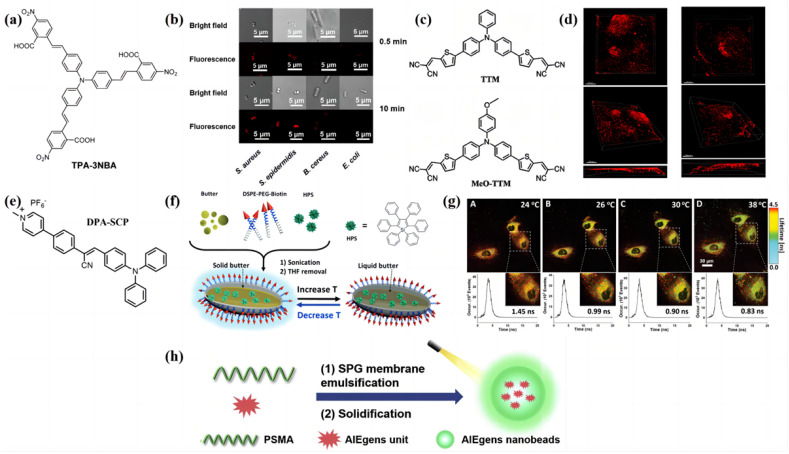
(**a**) Molecular structure of TPA-3NBA. (**b**) Confocal laser scanning microscopy (CLSM) images of *S. aureus*, *S. epidermidis*, *B. cereus,* and *E. coli* after 0.5 min or 10 min of incubation with TPA-3NBA. Adapted with permission from [69]. (**c**) Molecular structures of TTM and MeO-TTM. (**d**) CLSM images of atherosclerotic plaques; 3D images with different view angles of TTM-stained (left) and MeO-TTM-stained (right) atherosclerotic plaques. Scale bar = 200 μm. Adapted with permission from [70]. (**e**) Molecular structure of DPA-SCP. (**f**) Schematic diagram of the synthetic process of the HPS/butter/DSPE-PEG-biotin nanorod. Alterations of the position of HPS molecules and the orientation of the DSPE-PEG-biotin segment are not shown and the size of the nanorod is not drawn to scale. DSPE-PEG-Biotin, 1,2-distearoyl-snglycero-3-phosphoethanolamine-N-(biotinyl(polyethylene glycol)-2000) biotin; HPS, hexaphenyl-1H-silole; T, temperature. Adapted with permission from [72]. (**g**) In vitro fluorescence lifetime images and the corresponding fluorescence decay curves of the HPS/butter/DSPE-PEG-biotin nanorod in live HUVEC cells measured at the indicated temperature. Fluorescence lifetimes are displayed in a pseudocolor format. Excitation wavelength: 375 nm. Adapted with permission from [72]. (**h**) Schematic illustration for the preparation of AIE-gens nanobeads. Adapted with permission from [73]. The important parameters of the AIE material design strategy based on the functional groups are shown in Table 1.

In 2022, Chen’s group [69] designed a molecule named TPA-3NBA, which contains the TPA as the core and the styrene with nitro and carboxyl units as the arm chain (Figure 5a). They found that TPA-3NBA can selectively and rapidly stain (within 0.5 min) and effectively kill Gram-positive cocci without affecting bacilli under white light irradiation. In addition, this selective bacterial imaging approach enhances the material’s potential to quickly identify the local bacterial infection. Interestingly, the antibacterial activity can be further improved, since the normal concentration of divalent ions (Ca^2+^) in the human body can significantly increase the absorption of TPA-3NBA by Gram-positive cocci (Figure 5b). TPA-3NBA is a potential application prospect in targeted antimicrobial therapy. There are many probes that can kill the cocci. However, most of reported probes cannot exhibit the process when it comes to killing cocci. As a consequence, it is crucial for cocci-killing photosensitizers to combine the indicating and cocci-killing functions. Zhang et al. [70] employed TPA as the core and 2-cyanoethylene-thiophene as the arm when designing TTM and MeO-TTM (Figure 5c), which can be used for the lipid-droplet-specific bioimaging of cells and atherosclerosis plaques due to their strong AIE properties. The authors used both molecules to stain the ApoE^-/-^ mice. They found that the probes can not only brighten the lipid droplets (LDs) in AS plaques with low background noise but can also identify the spatial distribution of the LDs and can be used to image the depths of AS plaques (Figure 5d). TPA has also been used in functional groups introduced as side chains in sensor designs. Ding’s group [71] reported on small molecules containing TPA (Figure 5e), which had a significant germicidal effect on *E.* faecalis suspensions and 21-day-old biofilms in human root canals.

Except for the TPA, the HPS is also popularly introduced when designing high-performance AIE materials. The pH of the human body will fluctuate with the changes in body temperature. This factor restricts many fluorescent molecules from probing the body’s temperature. Gao et al. [72] reported on a nano-thermometer containing HPS as the AIE dye and household butter as the matrix (Figure 5f). The author used these nano-thermometers for temperature sensing in living HUVEC cells with fluorescence lifetime imaging microscopy (Figure 5g). They found that the nanosensors both inside and outside the HUVEC cells showed a long fluorescence lifetime (1.45 ns) at 24 °C (low temperature), with a redder color. However, once the temperature was increased to 38 °C, a shorter lifetime of 0.83 ns with a greener color was detected. The fluorescence responses upon temperature changes are reversible and are independent of the environmental pH. Later, Wu et al. [73] used HPS as the AIE dye and obtained nanobeads (Figure 5h), which were applied in the biosensor to detect the carcinoembryonic antigen concentrations in the cells. Therefore, early-age cancer can be easily detected. In addition, the long-term stability and toxicity are both essential factors in cancer detection.

Introducing functional groups with the ability to restrict intra-molecular motion is a simple and useful strategy in the design of AIE biosensors. Developing novel groups or units with these kinds of functionality is desirable and challenging, which is important for the further development of AIE biosensors.

## 3. Hydrogen Bonding and D-A System Strategy

Employing functional groups to reduce the intra-molecular motion in the aggregate state is a useful and simple method to design AIE biosensors, which has been widely studied by the scientific in the past few years. Promoting the commercialization of AIE biosensors and developing novel strategies to design high-performance AIE biosensors are important and urgent tasks. Introducing the bond with an electrostatic action within a single molecule or between the neighboring molecules may be a new strategy, since it might result in molecules with bright fluorescence instead of non-radiative decay through resisting the bonded groups’ motion in the aggregate state. Hydrogen bonding association with electrostatic action, whereby the bond force size between the chemical bond and van der Waals force has unique properties, is currently used to design AIE molecular systems.

**Figure 6:**
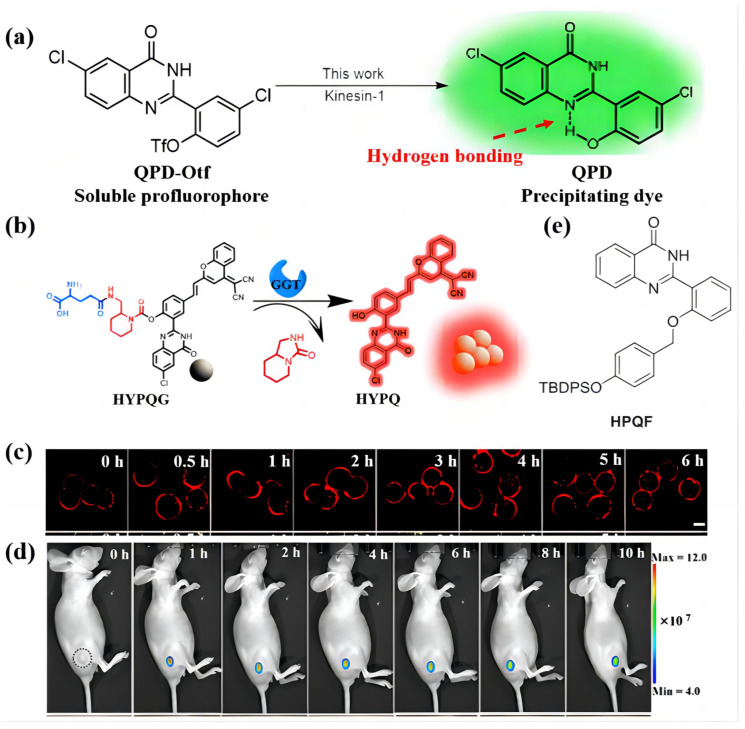
(**a**) The molecular structure of QPD-Otf and QPD. (**b**) The chemical structure and photophysical properties of HYPQG. Adapted with permission from [75]. (**c**) The long-term in situ images of HYPQG. Adapted with permission from [75]. (**d**) The long-term in situ imaging of GGT in mouse tumors. In vivo real-time imaging of GGT in A2780-bearing nude mice after tumor injection of 20 μM HYPQG. Adapted with permission from [75]. (**e**) The molecular structure of HPQF.

Angerani et al. [74] designed the QPD-OTf dyes shown in Figure 6a. Hydrogen bonds (N…HO) could be formed between the amide group and the hydroxyl units within the QPD core, which resist the two bonded groups’ vibration, resulting in a chromophore with AIE properties. The authors found that the QPD-Oft dyes could be used for kinesin-1 activity recordings in living cells. The premise of monitoring the native kinesin-1 transport activity is to form a regular crystal system, while the intramolecular hydrogen bond in QPD-OTf promotes the formation of the crystal system. QPD-OTf staining is compatible with live-cell imaging, which might be caused by the crystals dissolved in cell media after staining providing a non-destructive method to visualize the motion of kinesin-1 on Golgi-derived MTs.

QPD is one of the most used chromophores in the design of hydrogen-bonded AIE biosensors. In most cases, it exhibits a blue emission color. However, the biosensors emitting NIR light are more favorable, since the NIR light exhibits weak energy levels without causing biological tissue damage problems. Recently, some probes have been commercialized, such as allophycocyanin (APC) and Memb-Tracker Red. However, there are still some shortcomings that need to be solved, such as the diffusion phenomenon, which notably weakens the AIE appearance. Li et al. [75] used QPD units combined with the 2-(2-methyl–4H-chromen–4-ylidene) group to synthesize a series of dyes named HYPQ (Figure 6b). The hydrogen bonding enhances the link between HYPQ molecules. These dyes exhibit various emission colors ranging from blue to orange, and finally to red and even NIR. The authors used HYPQ dyes to image the A2780 cells in different regions. The fluorescence signal on the cell surface maintains the original shape for 6 h (Figure 6c). Furthermore, the long-term in situ imaging ability of HYPQG in tumor-bearing mice was investigated, which showed that the HYPQG probe could clearly distinguish the tumor from normal tissues, even after injection for 10 h. This indicated that the HYPQ could be used for the long-term in situ imaging of the tumor region (Figure 6d). In addition to the long-term imagining of the tumor region, Zhao et al. [76] combined the QPD group and tertbutyl diphenyl silane (TBDPS) unit to construct a molecule for the detection of fluoride ions in living cells (Figure 6e). However, the poor solubility was still a huge hindrance.

**Figure 7:**
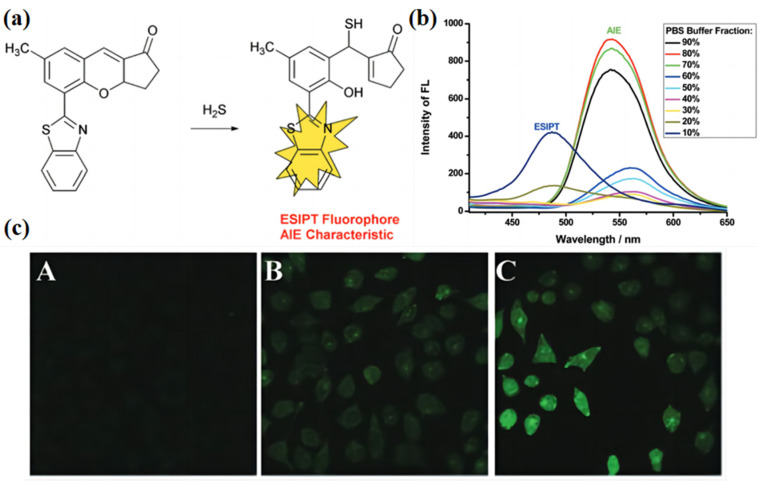
(**a**) Molecular structure of probe 1 and the proposed sensing mechanism for H_2_S. (**b**) Fluorescence spectra of probe 1 upon the addition of H_2_S (3 equivalents) in DMF with different vol% fractions of PBS buffer. Adapted with permission from [77]. (**c**) HeLa cells were incubated with 10 μM of probe 1 for 30 min, washed with DPBS, and incubated with (A) 0, (B) 10, and (C) 20 μM of H_2_S for 30 min each. Adapted with permission from [77].

Chen et al. [77] developed a chromophore containing 2-(2′-hydroxyphenyl)benzothiazole (HBT) combing AIE properties and color-changing characterization. The chemical structure of these dyes is shown in Figure 7a. The original dye presents weak emission in the solution state. Once it reacts with the hydrogen sulfide, the ring of this dye is opened; meanwhile, a hydroxyl group emerges (Figure 7a). The hydroxyl unit can react with the amide units from the benzothiazole core to form hydrogen bonds, which resist the benzothiazole motion, resulting in increased emission intensity. In addition, due to the change of the chemical structure, the emission wavelength is also changed from 480 nm to 540 nm (Figure 7b). The authors used this dye for the fluorescence imaging of H_2_S in HeLa cells. They found that the fluorescence from the cells was weak in the absence of additional H_2_S conditions (Figure 7c(A)), while the fluorescence became stronger and stronger once it was treated with more H_2_S (Figure 7c(B,C)). Changing chemical structure to format the hydrogen bond associations is a useful strategy to design “turn-on” fluorescent AIE biosensors.

Introducing hydrogen bonding into the molecules is a useful strategy in the design of AIE biosensors. Until now, most hydrogen bonds from the reported biosensors have been formed within single molecules. The development of chromophores with inter-hydrogen bonding might be another method to obtain AIE biosensors. In recent years, some electrostatic actions, such as halogen bonding, F…S interactions, N…S interactions, and so on, functioning as conformational locks, have been widely used in the design of materials with planar backbones to increase the charge transport mobility. This strategy might be suitable for AIE biosensors, since it can be used to resist molecule rotation or vibration.

For the purpose of designing high-performance fluorescent AIE materials, many researchers employ electron-rich and electrondeficient units in one single molecule to form a donor (D)-acceptor (A) system, since the fluorescence is often a resonance energy transfer effect and an intramolecular charge transfer effect. Through adjusting the donor and acceptor ability, the lowest non-occupied molecular orbital (LUMO) and the highest occupied molecular orbital (HOMO) levels of the molecules could be changed, thereby adjusting the emission color. When the identification group of the fluorescent probe is combined with the identified molecule, the change in the conjugated system leads to a change in the pull–push effect of the charge in the original molecule. This results in the charge transfer being weakened or enhanced, corresponding to a spectral red-shift or blue-shift being detected.

**Figure 8:**
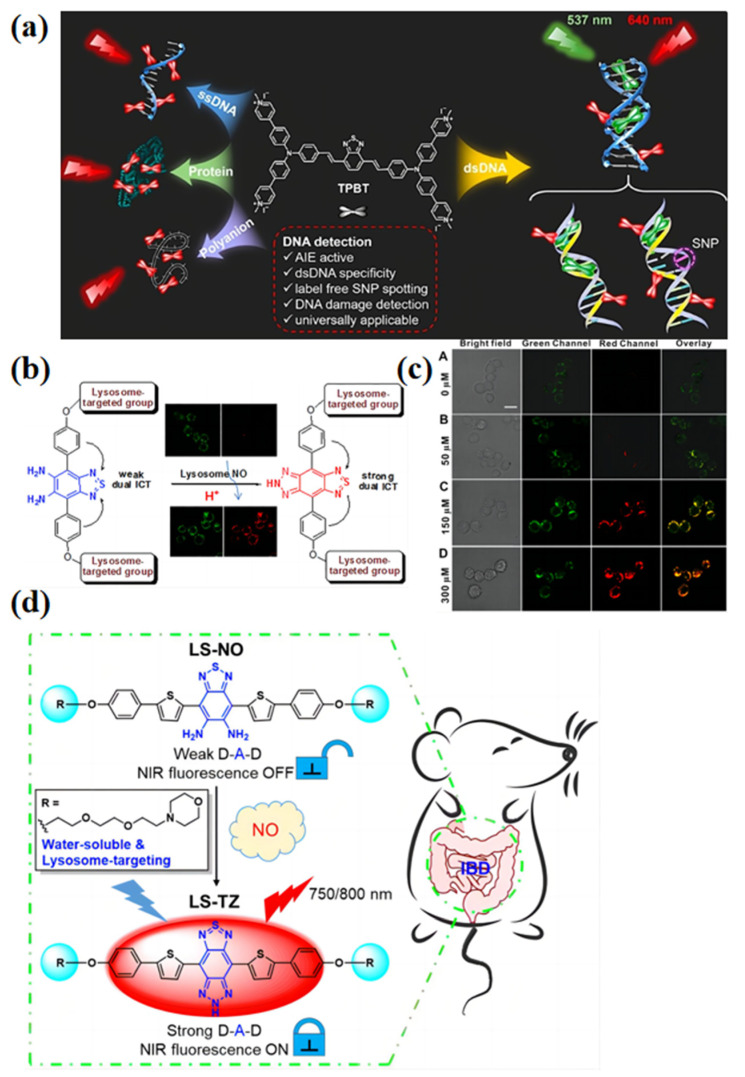
(**a**) Molecular structure of TPBT and its various emissions upon binding with different polyanionic macromolecules. Adapted with permission from [78]. (**b**) Molecular structure of MBTD. Adapted with permission from [79]. (**c**) CLSM imaging of RAW 264.7 cells treated with MBTD at various DEANO concentrations (0, 50, 150, 300 μM) for 1 h. Adapted with permission from [79]. (**d**) Schematic illustration of a water-soluble D−A−D-type NIR fluorescent probe named LS-NO for real-time detection of NO in the IBD model in vivo. Adapted with permission from [80].

Gao et al. [78] designed a D-A-D molecule with a strong AIE effect, named the TPBT (Figure 8a). The TPBT exhibited specificity in recognizing double-stranded DNA (dsDNA) by emitting a unique dual-color (red: ~649 nm; green: ~537 nm) fluorescent signal. The TPBT exhibits quite weak fluorescence at intensities around 640 nm, showing a red color in water and in the presence of single-stranded DNA (ssDNA), bovine serum albumin (BSA), or other polyanionic macromolecules. A new emission peak at around 537 nm appeared when it interacted with dsDNA via groove binding between the TPBT and dsDNA, resulting in the twisted intramolecular charge transfer (TICT) effect. Thus, this D-A-D chromophore has the potential for specific and label-free double-stranded DNA recognition and single-nucleotide polymorphism detection.

Besides the interaction between the AIE biosensor with the cells resulting in the ICT effect, the chemical structure change might also produce a signal for the detection process. Wang et al. [79] designed a D−A−D type near-infrared (NIR) fluorescent probe named MBTD, and used it for the detection of nitric oxide (NO) in vivo and ex vivo. The MBTD exhibits a weak dual-ICT effect without NIR fluorescence. However, it can react with NO in the lysosomes of living cells; meanwhile, a new MBTT molecule with a strong dual-ICT effect is formed with a strong NIR emission (Figure 8b). The authors found that the cells displayed a green fluorescence after 1 h of incubation with MBTD in the absence of exogenous NO conditions. The green fluorescence emission of the MBTD colocalized well with the red fluorescence signal of LysoTracker Red but not MitoTracker Red. With 50 μM of DEANO (nitric oxide (NO) donor) added, the NIR fluorescence was observed, while the emission became stronger with increasing amounts of DEANO (NO donor) (Figure 8c). Later, Liu [80] designed a similar chromophore named LS-NO for the real-time detection of NO in inflammatory bowel disease (IBD) by harnessing the enhanced intramolecular charge transfer mechanism (Figure 8d). LS-NO has great potential as a long-time imaging agent for the real-time detection of NO in vivo, distinguishing IBD mice from normal mice simultaneously. In long-term imaging, stability surely is an important factor, but we should concentrate more on the comprehensive influence on human health of the long-term imaging molecules.

**Figure 9:**
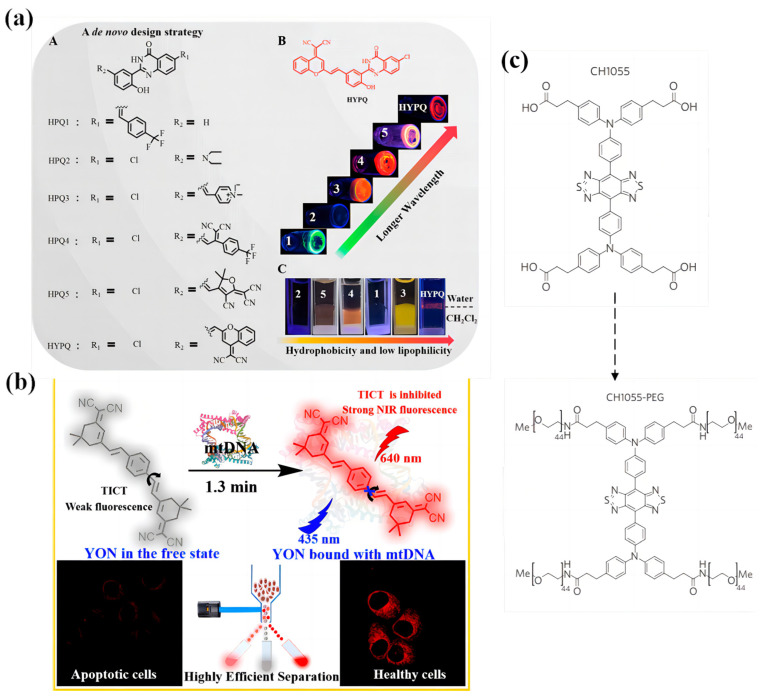
(**a**) The design of hydrophobicity and low lipophilicity NIR solid-state fluorochrome. (A) A de novo design strategy for developing solid-state fluorophores. (B) Solid-state fluorescent photographs of those fluorophores in the powder samples. (C) Diffusion experiments were conducted at the interface between water and dichloromethane (amphiphilic environment). All fluorescent photos were obtained under UV lamp excitation at 365 nm. Adapted with permission from [75]. (**b**) The binding of YON to mtDNA. Adapted with permission from [82]. (**c**) The chemical structure of CH1055 and the one-step synthesis of CH1055-PEG.

The far-red region or the NIR emission biosensor is favorable, since it can avoid endogenous fluorescence interference from the organism, resulting in minimal cell damage and exhibiting enhanced tissue penetration ability [81]. In addition, these kinds of biosensors might be used for two-photon absorption techniques for imaging. The two two-photon absorption technique often uses light with a long wavelength and weak energy, which causes no damage to tissues or cells. Such biosensors might be designed by employing strong donor and acceptor groups to form a strong D-A, D-D, or A-A system, since these systems could increase the material’s HOMO energy level or decrease the LUMO energy level, resulting in red or NIR emissions. Li et al. [75] designed a series of chromophores using the HPQ group combined with various units with different donor and acceptor abilities. The authors found that the emission color of these chromophores could be adjusted steeply into long wavelengths from blue to orange and finally to NIR (Figure 9a(A,B)). Moreover, HYPQ shows better antidiffusion properties than HPQ1-5 (Figure 9a(C)). Recently, Wang et al. [82] reported on an A-π-D-π-A-type molecule called YON based on the chromophore of the electron-deficient unit dicyanoisophorone, which exhibits strong NIR fluorescence at 640 nm (Figure 9b). As the AIE biosensor, the YON could specifically bind mtDNA in living cells with far-NIR fluorescence, while it exhibited almost no fluorescence in the apoptotic cells. This was because after binding with mtDNA, the YON molecule switched from the opposite side to the same side of the molecule, which greatly reduced the intramolecular dipole moment as well as the bandgap, thereby enhancing the ICT effect and improving the sensitivity for mtDNA detection. As a consequence, a very sensitive fluorescence response was observed. Compared to the NIR biosensors, the biosensor with the second near-infrared window (NIR-II) of fluorescence is more favorable, since it can be used to image tissues at centimeter depths and achieve micrometer-scale resolution at depths of millimeters. Antaris et al. [83] designed a D-A-D molecule named CH1055 (Figure 9c), which exhibits a peak fluorescent emission at ∼1055 nm. The author successfully used it to image the blood vasculature, lymphatic vasculature, and tumors. In addition, we have seen many toxicity experiments on fluorescence probes with mice, but clinical experiments are truly decisive for guiding targeted tumor resection. Table 2 shows the important parameters for hydrogen bonding and the D-A system strategy in AIE probes.

## 4. Conclusions and Outlook

AIE molecules, exhibiting strong fluorescence in the aggregate state, overcome the ACQ effect as compared to traditional fluorescent dyes. Due to their specific fluorescence properties, they are widely used in biosensors to label some special metal ions, gases, tissues, and cells from the human body or animal bodies. The development of AIE biosensors could help in predicting or checking physical health, and could further significantly improve human living and medical standards, as well as extending the lifetime. For the purpose of obtaining such high-performance AIE biosensors, the molecular design concept plays a crucial role. The introduction of functional chromophores with AIE properties is a simple and useful strategy for the design of AIE biosensors. These kinds of functional chromophores mostly present properties with restrictions on the rotation or vibration of some parts from the molecules, such as triphenylamine, hexaphenylsilole, tetraphenylethylene, and biphenylvinyl anthracene. Besides the functional groups, the dyes with intra-hydrogen bonding associations are also potentially used in the AIE biosensors, since they resist the bonded groups’ motion in the aggregate-state properties. To obtain far-NIR or NIR AIE biosensors, the D-A system is often used, since it can reduce the molecule band gap, resulting in a red-shift in fluorescence. 

Although AIE fluorescent probes have been developed for many years and have been used in the biomedical field, this field is still in its early infancy. For the further development of high-performance AIE biosensors and the promotion of their commercialization, there are still many issues with AIE fluorescent probes that should be addressed: (i) it is important to identify whether the sensors will affect the physiological activities of the organisms; (ii) the water solubility of AIE fluorescent probes is an important problem that currently limits their application and development; (iii) many luminescence mechanisms of various AIE fluorescent probes are still vague; (iv) the high-performance AIE biosensors combine multiple advantages into one molecule, such as strong fluorescence in the NIR or NIR-II region, good stability, and biocompatibility with non-toxicity; (v) more applications should be further developed.

## Figures and Tables

**Table 1:** The important parameters of the AIE material design strategy based on the functional groups.

Probe	Cell-Imaging or Detection	Emission Wavelength (λ_em_) (nm)	Excitation Wavelength (λ_ex_) (nm)	Ref.
TPE-SH4	Dithiothreitol/Trifluoroacetic acid	478	390	[58]
PBI-TPE-11	HeLa cells imaging	660	575	[59]
Probe L	Zn^2+^ in HeLa cells	484	416	[60]
HA-Ac-Pha	cancer cells	460	312	[61]
NDSA	A549 cells imaging	554	456	[68]
CNDSA	A549 cells imaging	542	453	[68]
TTM	lipid droplets	684.2	575.2	[70]
MeO-TTM	lipid droplets	719.2	684.2	[70]
HPS/butter/DSPE-PEG-biotin	fluorescent thermometer	490	370	[72]

**Table 2:** The important parameters for hydrogen bonding and the D-A system strategy in AIE probes.

Probe	Detection or Cell-Imaging	Emission Wavelength (λ_em_) (nm)	Excitation Wavelength (λ_ex_) (nm)	Ref.
HYPQG	imaging of γ-glutamyltranspeptidase activity on the cell membrane	584–676	488	[75]
Probe 1	Hydrogen sulfide	540	350	[77]
TPBT	specifically recognize double-stranded DNA (dsDNA)	537	450	[78]
MBTD	lysosomal nitric oxide (NO)	565	365	[79]
LS-NO	NO in Inflammatory bowel disease	760/804	650	[80]
YON	Mitochondrial DNA	640	435	[82]

## Data Availability

Not applicable.
